# Lipid Rafts: Keys to Sperm Maturation, Fertilization, and Early Embryogenesis

**DOI:** 10.1155/2011/264706

**Published:** 2011-01-12

**Authors:** Natsuko Kawano, Kaoru Yoshida, Kenji Miyado, Manabu Yoshida

**Affiliations:** ^1^Division of Gamete and Reproductive Biology, National Research Institute for Child Health and Development, 2-10-1 Okura, Setagaya, Tokyo 157-8535, Japan; ^2^Biomedical Engineering Center, Toin University of Yokohama, Yokohama 225-8502, Japan; ^3^Misaki Marine Biological Station, Graduate School of Science, University of Tokyo, Miura, Kanagawa 238-0225, Japan

## Abstract

Cell membranes are composed of many different lipids and protein receptors, which are important for regulating intracellular functions and cell signaling. To orchestrate these activities, the cell membrane is compartmentalized into microdomains that are stably or transiently formed. These compartments are called “lipid rafts”. In gamete cells that lack gene transcription, distribution of lipids and proteins on these lipid rafts is focused during changes in their structure and functions such as starting flagella movement and membrane fusion. In this paper, we describe the role of lipid rafts in gamete maturation, fertilization, and early embryogenesis.

## 1. Introduction

Fertilization is the process in which 2 different gamete cells, a sperm and an oocyte, unite to produce a zygote. For fertilization to be successful, these gamete cells must differentiate and activate specific signaling pathways. For example, after sperm has differentiated completely, various extracellular factors such as epididymosomes and albumin alter the structure and function of the plasma membrane of the sperm. In addition, in terminally differentiated gamete cells, various sterols, sphingolipids, glycolipids, and glycosylphosphatidylinositol- (GPI-) anchored proteins are localized on cell membrane microdomains that are called lipid rafts. Lipid raft components are often examined by using detergent-resistant membrane domains (DRMs), which enrich these components so that their distributions and functions can be visualized on the cell surface by using putative raft markers [1, 2]. Since lipid rafts in gametes contain signaling proteins that regulate intracellular functions and cell signaling, these domains are important for sperm maturation, fertilization, and early embryogenesis [3, 4]. In this paper, we discuss the role of lipid rafts in reproductive biology.

## 2. Sperm Maturation and Membrane Modification

**Figure 1:**
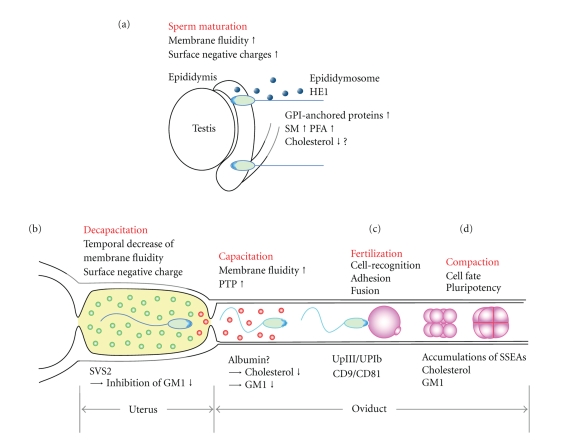
Schematic of lipid rafts in gamete formation, function, fertilization, and early embryogenesis. (a) Sperm mature, gaining motility and fertilizing abilities, during epididymis transit. The extracellular factors, epididymosome and HE1, dynamically change the components of the sperm plasma membrane. (GPI, glycosylphoshphatidylinositol; SM, sphingomyelin; PFA, polyunsaturated membranous fatty acids). (b) Ejaculated sperm are temporally bound to SVS2 (decapacitation). SVS2 binds to GM1 of the sperm head in the uterus, resulting in the inhibition of the fertilizing ability of sperm. Subsequently, the sperm that migrate to the oviduct undergo capacitation. Capacitation causes an efflux of cholesterol and G_M1_ from the plasma membrane and an increase of membrane fluidity and protein tyrosine phosphorylation (PTP). (c) Sperm recognize and adhere to UpIII/UpIb of Xenopus oocyte and fuse with CD9/CD81 of murine oocyte plasma membrane. These molecules are enriched in lipid rafts, and oocytes treated with cyclodextrin prevent the sperm from fertilization. (d) In early embryogenesis, SSEAs are colocalized with cholesterol and G_M1_ plays an important role in the compaction of an embryo, leading to the decision of cell fate and its pluripotency.

Sperm are highly differentiated haploid cells with a head and a tail (flagellum) [5]. The head consists of a nucleus, an acrosome, and a small amount of cytoplasm, while the tail consists of a motility apparatus, mitochondria, an axoneme, and cytoskeletal structures. Although these structures are necessary for sperm to swim and fertilize oocytes, these structures are not functional after spermatogenesis until the plasma membrane is modified during epididymal transit (Figure 1(a)) [6]. In mammals, the sperm mature in the epididymis; however, in other animals, sperms mature in the spermiduct [7]. Previous studies have demonstrated that the modifications of the sperm plasma membrane that occur during epididymal transit include changes in its lipid and protein composition, modifications of surface proteins, and increased total negative charge of the extracellular surface [8, 9].

Electron microscopy studies showed that the epididymal lumen contained membranous vesicles that which called epididymosomes [10–12]. These vesicles, which are particularly rich in sphingomyelin (SM) and arachidonic acids, are secreted via apocrine secretion. In addition, two-dimensional gel electrophoresis and liquid chromatography-quadrupole time-of-flight (LC-QToF) analyses show that epididymosomes contain endoplasmin (heat shock protein 90 *β*1; Hsp90*β*1), 70 kDa heat shock protein 5, chaperones, and other exosomes [13–16]. In addition, integral membrane proteins, such as GPI-anchored proteins, also are associated with epididymosomes. Several GPI-anchored proteins in epididymosomes have been found in the mature sperm of various animals. For example, HE5 (CD52) is found in human sperm [17], SPAM 1 and hyaluronidase are present in mouse sperm [18–21], and P26h [22] and P25b [23] are found in hamsters and bulls, respectively [24]. In addition, epididymosomes localize to the sperm membrane during epididymal transit and contribute to the formation of various membrane structures such as lipid rafts in sperm [25]. Furthermore, in sperm membrane lipids, the percentage composition of both SM and various polyunsaturated fatty acids, which are mainly arachidonic, docosapentaenoic, and docosahexaenoic acids, increases throughout the epididymal tract [12].

**Figure 2:**
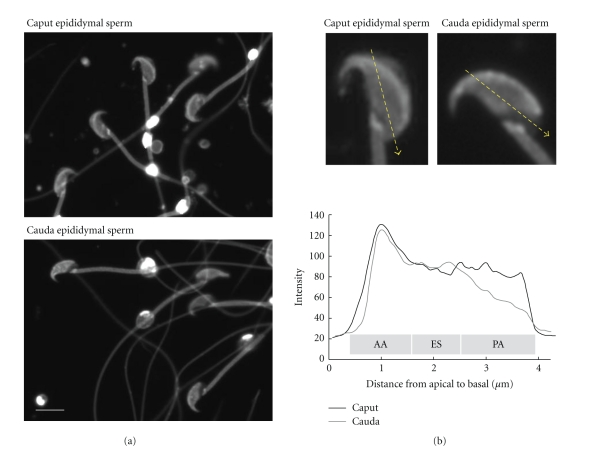
Distribution of cholesterol during sperm maturation in murine epididymis. (a) Sperm collected from caput epididymis reveal filipin signal on the whole head. After epididymal transit (cauda epididymis), the signal is not detected in the postacrosomal region (PA). Scale bar = 5 *μ*m. (b) Densitometric analysis shows a significant decrease of filipin signal at the postacrosomal region. AA, apical acrosome; ES, equatorial segment; PA, postacrosomal.

Six genes, known as *HE1−HE6*, are expressed specifically in the human epididymis [26–28]. Mutations in *HE1* cause Niemann-Pick type C2 (NPC2) disease, a fatal neurovisceral disorder that is characterized by the accumulation of cholesterol in lysosomes [29]. HE1 is a small, soluble glycoprotein with 132 amino acids that binds to cholesterol, but not to cholesterol derivatives that have hydrophilic substitutions on their isooctyl side chains [29–32]. Xu et al. [33] determined the X-ray crystallographic structure of bovine NPC2 protein complexed with cholesterol sulfate. Together, these studies showed that HE1 binds to cholesterol *in vitro* and may regulate the cholesterol content in sperm throughout the epididymal tract. However, other studies have demonstrated that the ratio of total phospholipid to total cholesterol does not change during epididymal transit [12, 34]. Previously, we used filipin as a cytochemical probe for membrane cholesterol in the sperm plasma membrane during epididymal maturation (Figure 2). Our results show that the filipin signal decreased at the post-acrosomal region during epididymal transit. Similar pattern of filipin was observed in boar sperm [35]. Previous studies have shown that the rate of pregnancy in humans decreases as the amount of time between a vasectomy and its reversal increases [36–38]. In addition, sperm from men who have undergone vasectomy reversal have higher levels of HE1, cholesterol, and ganglioside G_M1_ compared with sperm from fertile men who have not had vasectomy [39]. These findings suggest that HE1 regulates the amount or localization of sperm lipid rafts in the epididymis, which produces mature sperm.

Several studies have shown that modification of SM, polyunsaturated fatty acids, cholesterol, and G_M1_ changes the fluidity of sperm membranes and the composition of lipid rafts. For example, lipid diffusion in the plasma membrane of mouse sperm increased significantly during their transition from the caput epididymis to the cauda epididymis [40]. Moreover, Nishio et al. [41] reported that pheochromocytoma PC12 cells that overexpress G_M1_ did not exhibit any neurite formation, even after stimulation with nerve growth factor (NGF). Furthermore, increased expression of G_M1_ reduces membrane fluidity, disorders the lipid raft, and changes the intracellular localization of NGF receptors and related signaling molecules [41]. Further studies are needed to elucidate the mechanisms and molecules that promote sperm maturation in the epididymis.

## 3. Sperm Lipid Rafts and Fertility

After maturing in the epididymis, sperm are able to swim and fertilize an oocyte (Figure 1(b)). In invertebrates that reproduce by external fertilization, sperm are usually capable of fertilization immediately after they become motile. In contrast, in mammals that reproduce by internal fertilization, sperm are unable to fertilize oocytes immediately after they can swim. Instead, sperm acquire this ability after moving to the uterus and remaining for an appropriate period of time [42, 43]. The biochemical process that confers this ability to sperm is called “capacitation.” During sperm capacitation, protein tyrosine phosphorylation occurs along with hyperactivated flagellar beating; further, the acrosome reaction is induced, and the sperm penetrates the zona pellucida (ZP) and finally binds and fuses with the oocyte. In addition, the organization of membrane proteins and lipids changes significantly [44–48]. *In vitro,* capacitation requires a high concentration of albumin in culture medium to decrease the sperm cholesterol/phospholipid ratio [49, 50]. Fluorescent and electron microscopic studies have shown that a combination of bicarbonate and albumin promotes membrane distribution and cholesterol efflux [35, 51, 52]. Albumin also decreases the amount of sialic acid, ganglioside, and triglyceride in the sperm plasma membrane [49]. In addition, methyl-*β*-cyclodextrin (MBCD) promotes sperm capacitation *in vitro*, by decreasing the amount of cholesterol in the plasma membrane and disrupting lipid rafts; however, the critical concentration of MBCD should be considered [53–56]. The strong MBCD treatment also reveals some proteins that are involved in capacitation-dependent processes and ZP binding [57]. The physiological relevance of raft reordering in the sperm surface is to create protein complexes involved in ZP binding [58, 59].

Cholera toxin subunit B (CTB), which binds to ganglioside G_M1_ with a high affinity, has been widely used as a reporter of the distribution of lipid rafts and to study the CTB-binding pattern of sperm in both *in vitro* and *in vivo* conditions. We, along with several other research groups, also have developed methods to investigate the distribution of G_M1_ in fixed sperm. These studies have revealed that G_M1_ expression is significantly altered during sperm capacitation and the acrosome reaction [60–62]. In contrast, the CTB-binding pattern of sperm is variable and depends on their fixation conditions [63]. In living sperm, Selvaraj et al. [64] demonstrated that the cyclodextrin treatment does not change the distribution of G_M1_ in mouse or bovine sperm. However, the use of specific fixation conditions induced stimulus-specific patterns of G_M1_ distribution. Specifically, in mouse sperm, G_M1_ was broadly localized from the postacrosomal region to throughout the sperm head. Shadan et al. [61] showed that the distribution of G_M1_ in boar sperm changes sequentially, from the tail to the head, during MBCD-mediated capacitation. One of our previous studies also demonstrated the CTB- binding pattern in murine sperm in physiological conditions [62]. Briefly, ejaculated sperm were collected from the female mice when the sperm were first detected in their oviducts (approximately 3 hours after copulation). Their reproductive tracts were divided into 4 parts, namely, the oviduct, uterine region near the oviduct, uterine region near the cervix, and vagina. While the sperm migrated from the uterus to the oviduct, CTB fluorescence was lost from the postacrosomal region. In addition, G_M1_ interacted with seminal vesicle secretion 2 (SVS2), which is secreted from seminal vesicles and inhibits sperm capacitation. Since SVS2 is a highly basic protein, this interaction depends on its charge. In addition, CTB also inhibited sperm capacitation. Increasing evidence suggests that sperm capacitation is regulated by the distribution of G_M1_ or its charge on the sperm plasma membrane. However, staining pattern by CTB is not consistent with that by other probes, such as lysenin and antibody against Thy-1.2 [65, 66]. Lysenin and the antibody have high affinities for sphingomyelin and GPI-anchored protein enriched in the DRM fractions, respectively [65]. Further investigation is needed to elucidate the localization of lipid rafts more precisely.

Some lipid rafts are found in cell surface invaginations called caveolae. These invaginations are formed from lipid rafts by polymerization of caveolins, which are palmitoylated integral membrane proteins that bind to cholesterol with high affinity [67, 68]. Previous studies have demonstrated that caveolin-1 and -2 are enriched in the Triton X-100-insoluble membrane fraction of mature sperm and localize to the acrosomal membrane [69, 70]. Since these caveolins disappear after the completion of the acrosome reaction, they are thought to regulate the acrosome reaction. However, caveolin-1-and -2-null mice are fertile, and the distribution of G_M1_ in caveolin-1-deficient mouse sperm is comparable to that in wild-type mouse sperm [63, 71, 72]. These findings suggest that caveolae are not required for the presence of lipid rafts in the acrosomal membrane and fertilization of mouse sperm. However, several other studies indicate that lipid rafts in the apical ridge head area of sperm have affinity for the ZP [57, 59]. Since the ZP is involved in the acrosome reaction, the function of lipid rafts in this reaction is still controversial.

## 4. Oocyte Lipid Rafts and Fertility

In general, sex hormones stimulate the continuation of the first meiotic division of oocytes. However, in some animals, this occurs after the oocyte is released from an inhibitory environment. In both vertebrates and invertebrates, the first meiotic division of oocytes is asymmetric, which results in the formation of a relatively large oocyte and small polar bodies. During meiotic arrest, oocytes are fertilized by sperm, and then the second meiotic division is completed. The activation of oocytes upon fertilization is a Ca^2+^-dependent process in all animals [73, 74]. Furthermore, intracellular Ca^2+^ ([Ca^2+^]*_i_*) is a key regulator of many cellular functions [75]. In oocytes, increased [Ca^2+^]*_i_* stimulates the continuation of the second meiotic division and formation of the second polar body, followed by formation of male and female pronuclei [47, 75]. The signaling pathway that regulates this increase in [Ca^2+^]*_i_* is highly conserved among species [76]. In this pathway, phospholipase C-(PLC-) dependent production of inositol 1,4,5-triphosphate (IP_3_) triggers the increase in [Ca^2+^]*_i_*, which propagates from the endoplasmic reticulum. In contrast, the binding of sperm to the oocyte plasma membrane is species specific. Currently, there are 2 hypotheses about how a sperm binds and activates an oocyte, namely, a transmembrane receptor mechanism that involves G proteins and a soluble sperm factor mechanism [77].* Xenopus *oocytes have a G protein-coupled sperm receptor that activates PLC on the plasma membrane (Figure 1(c)). In addition, lipid-raft-associated proteins, such as uroplakin III (xUPIII) and its tetraspanin-binding partner uroplakin Ib (UPIb), are involved in the sperm-oocyte membrane interaction and subsequent oocyte activation. Specifically, xUPIII is cleaved by sperm protease and promotes oocyte activation via Src tyrosine kinase and PLC*γ* signaling [76, 78, 79]. xUPIII and UPIb form a complex on the oocyte plasma membrane and colocalize with G_M1_ [80]. In addition, CD9, a tetraspanin that is involved in the sperm-oocyte fusion in mice, is found in the DRM fraction of *Xenopus* oocytes; however, CD9 does not interact with xUPIII or UPIb [80]. These findings suggest that the mechanism of sperm-oocyte fusion in *Xenopus* is different from that in mouse, although lipid rafts are involved in this process in both species.

**Figure 3:**
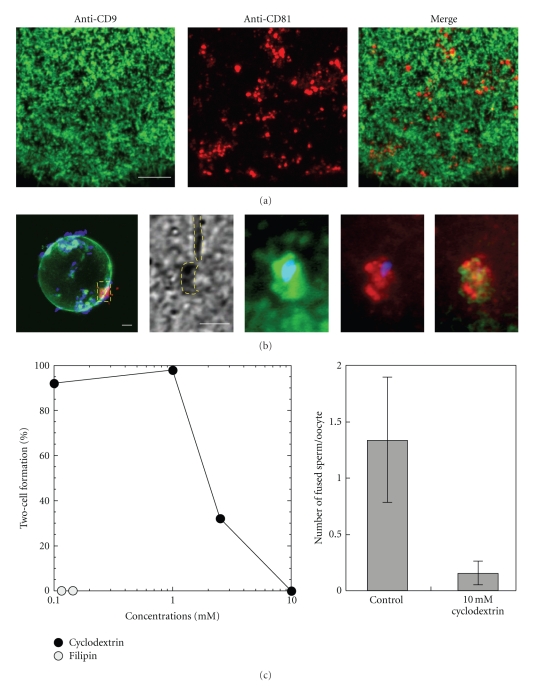
Distribution and function of CD9 and CD81 in murine oocyte. (a) The distribution of CD9 (green) is distinct from that of CD81 (red). CD9 localizes over the entire surface membrane, except for the MII plate, whereas CD81 shows patches with a low frequency. Scale bar = 10 *μ*m. (b) Cell surface-bound sperm shows colocalization of CD9 and CD81 with sperm nuclei (blue). Scale bar = 10 *μ*m. (c) Pretreatment of an oocyte with cyclodextrin (CD) prevents sperm from fusing and fertilizing the oocyte. The same phenomenon is observed in the treatment of filipin.

Furthermore, 2 plasma membrane proteins, CD9 and CD81, are important molecules in murine sperm-oocyte fusion [81, 82]. CD9 and CD81 belong to the tetraspanin superfamily and form a complex with integrin *α*3*β*1; moreover, CD63 that forms a complex with CD9 and CD81 localize- along with phosphatidylinositol 4-kinase within lipid raft-like microdomains in A431 and HT1080 cell lines [83]. Female CD9 knockout mice are infertile; although they produce oocytes that mature normally, these oocytes cannot fuse with sperm [81]. CD81, which has a similar structure and function as CD9, also is involved in gamete fusion. However, the effects of deleting CD81 are less dramatic than those of deleting CD9 [82, 84]. The expression level of CD9 is not affected by a deficiency of CD81 in murine oocytes and vice versa. Both CD9 and CD81 are localized on the surface of murine oocytes (Figure 3(a)). However, their distributions were completely different. As previously described [85], CD9 was expressed on the oocyte microvilli. Whereas CD81 was distributed in microdomain-like structures between microvilli that expressed CD9. However, both CD9 and CD81 were concentrated at the sperm attachment site (Figure 3(b)). Cyclodextrin, a lipid-raft disruptor, inhibited sperm-oocyte fusion and decreased the percentage of two-cell formation in a dose-dependent manner (Figure 3(c)). Similarly, filipin had the same effect on the oocyte plasma membrane. As a result, it is likely that CD9 and CD81 are important for coordinating the sperm-oocyte fusion process in mice.

## 5. Lipid Rafts in Early Development

After fertilization, the zygote divides into a blastocyst that can be implanted into the uterus in mammals. At the 8-cell stage, a murine embryo undergoes compaction to form polarized morulae (Figure 1(d)). This process involves substantial changes in cellular organization. Consequently, positional and functional differences occur among the blastocyst cells [86]. FILIA-MATER complexes localize asymmetrically in the apical cytocortex of 2-cell embryos due to their absence near cell-cell contact [87]. Although this asymmetry is reversible when the blastomeres of 2- and 4-cell embryos are separated, FILIA-MATER complexes are detected at the apical subcortex of “outer” but not “inner” cells of morulae. These findings indicate that the plasticity of the localization of FILIA-MATER complexes may reflect the cell fate determination of preimplantation mouse embryos. The outer cells of the morulae become the mural trophectoderm while the inner cells form the inner cell mass (ICM) of the blastocyst. Originally, embryonic stem (ES) cells were isolated from the ICM of the blastocyst and were shown to be pluripotent and self-renewing. The studies about FILIA-MATER complexes and ES cells suggest that cell-cell adhesion that results from compaction may be responsible for pluripotency.

Many cell-surface antigens that are markers of pluripotency have been identified in the ICM, ES cells, and embryonic carcinoma cells. The most common marker of murine ES cells is stage-specific embryonic antigen (SSEA)-1. The expression of this antigen changes dramatically in preimplantation mouse embryos. Specifically, SSEA-1 is highly expressed in the morula stage, suppressed after compaction, and then expressed only in the ICM of the blastocyst [88]. The Lewis^x^ epitope of SSEA-1 (Gal*β*1 → 4(Fuc*α*1 → 3)-GlcNAc*β*1 → 3Gal) also is found on glycosphingolipids and glycoproteins [89–91]. Mouse blastomeres and embryonic carcinoma cells identify each other and aggregate by recognizing this epitope [92, 93]. Similarly, SSEA-3 and -4 are common markers of human pluripotent stem cells and are highly expressed before the morula stage but decline afterwards [94]. The SSEA-3 and -4 epitopes are unique globo-series glycosphingolipids, namely, R-3GalNAc*β*1 → 3Gal*α*1 → 4R^'^ and NeuAc*α*2 → 3Gal*β*1 → 3-GalNAc*β*1 → 3Gal*α*1 → 4R^'^, respectively [95–97]. The SSEA-4 epitope is found in the lipid rafts of ACHN, a human renal cancer cell line [98]. In viable murine embryos, an anti-SSEA-4 antibody detects SSEA-4 over the entire membrane surface, with some accumulation at the interface between blastomeres [99]. A similar pattern of localization also was also observed with CTB staining [100]. Because other SSEAs are homologous to glycosphingolipids and share similar chemical properties, they are also likely to be enriched in lipid-rafts. SSEA-1 is colocalized with adhesion-related proteins, such as CD9, ICAM-1, and PECAM-1, in the contact regions of murine embryos and ES cells [101]. These proteins also are lipid raft-associated proteins [102–104] that are distributed in a way similar to SSEA-4. Collectively, these findings suggest that SSEA-1, -3, -4 are localized in lipid rafts, are involved in cell-cell adhesion, and may contribute to the pluripotency of mouse ES cells.

In addition to cell adhesion, lipid rafts play an important role in cytokinesis [105], which is a complex process that involves dynamic cortical rearrangement. Surprisingly, clathrin mutations that affect endocytosis cause defects in cytokinesis in many organisms [106, 107]. Feng et al. [108] showed that clathrin and caveolae are localized at the cleavage furrow in zebrafish blastomeres. In embryos, MBCD inhibits endocytosis and prevents normal cytokinesis. Ganglioside G_M1_, cholesterol, and tyrosine-phosphorylated proteins also have been found in the cleavage furrow and plane of sea urchin embryos [109]. In these embryos, DRMs contain Src and PLC*γ*, which are tyrosine phosphorylated at the site of cytokinesis. Furthermore, activation of these enzymes is required for furrow progression. These studies suggest that caveolae and lipid rafts contribute to cytokinesis in early developmental embryos.
